# Frequency of human immunodeficiency virus (HIV) testing in urban vs. rural areas of the United States: Results from a nationally-representative sample

**DOI:** 10.1186/1471-2458-11-681

**Published:** 2011-09-01

**Authors:** Michael E Ohl, Eli Perencevich

**Affiliations:** 1 VA Office of Rural Health (ORH), Veterans Rural Health Resource Center-Central Region, Iowa City VAMC, Iowa City, IA, USA; 2 Center for Comprehensive Access and Delivery Research and Evaluation (CADRE), at the Iowa City VA Medical Center, Iowa City, IA, USA; 3 Department of Internal Medicine, University of Iowa Carver College of Medicine, Iowa City, IA, USA

## Abstract

**Background:**

Studies in the United States show that rural persons with HIV are more likely than their urban counterparts to be diagnosed at a late stage of infection, suggesting missed opportunities for HIV testing in rural areas. To inform discussion of HIV testing policies in rural areas, we generated nationally representative, population-based estimates of HIV testing frequencies in urban vs. rural areas of the United States.

**Methods:**

Secondary analysis of 2005 and 2009 Behavioral Risk Factor Surveillance System (BRFSS) data. Dependent variables were self-reported lifetime and past-year HIV testing. Urban vs. rural residence was determined using the metropolitan area framework and Urban Influence Codes and was categorized as 1) metropolitan, center city (the most urban); 2) metropolitan, other; 3) non-metropolitan, adjacent to metropolitan; 4) non-metropolitan, micropolitan; and 4) remote, non-metropolitan (the most rural).

**Results:**

The 2005 sample included 257,895 respondents. Lifetime HIV testing frequencies ranged from 43.6% among persons residing in the most urban areas to 32.2% among persons in the most rural areas (P < 0.001). Past-year testing frequencies ranged from 13.5% to 7.3% in these groups (P < 0.001). After adjusting for demographics (age, sex, race/ethnicity, and region of residence) and self-reported HIV risk factors, persons in the most remote rural areas were substantially less likely than persons in the most urban areas to report HIV testing in the past year (odds ratio 0.65, 95% CI 0.57-0.75). Testing rates in urban and rural areas did not change substantively following the 2006 Centers for Disease Control and Prevention recommendation for routine, population-based HIV testing in healthcare settings. In metropolitan (urban) areas, 11.5% (95% CI 11.2-11.8) reported past-year HIV testing in 2005 vs. 11.4% (95% CI 11.1%-11.7%) in 2009 (P = 0.93). In non-metropolitan areas, 8.7% (95% CI 8.2%-9.2%) were tested in 2005 vs. 7.7% (95% CI 7.2%-8.2%) in 2009 (P = 0.03).

**Conclusions:**

Rural persons are less likely than urban to report prior HIV testing, which may contribute to later HIV diagnosis in rural areas. There is need to consider strategies to increase HIV testing in rural areas.

## Background

Over the past three decades the human immunodeficiency virus (HIV) epidemic in the United States (US) has slowly but persistently spread from large cities to involve more rural areas. The Centers for Disease Control and Prevention (CDC) now estimate that 8-9% of persons with newly diagnosed Acquired Immune Deficiency Syndrome (AIDS)-an advanced stage of HIV infection-reside in non-metropolitan (rural) areas [1]. In comparison, 17% of the US population resides in non-metropolitan areas [2].

Rural persons with HIV are more likely than their urban counterparts to be diagnosed and enter care at advanced stages of infection [3,4]. This is significant because later diagnosis is associated with both worse outcomes and increased HIV transmission [5]. Later HIV diagnosis in rural areas suggests that rural persons are less likely to have an HIV test during the early, generally-asymptomatic years of infection, and indicates a need for strategies to increase HIV testing in rural populations.

An analysis of National Health Interview Survey (NHIS) data from 1991-prior to the availability of effective HIV therapy-found that rural persons were less likely than urban to report prior HIV testing [6]. However, there are no published reports of the frequency of HIV testing in urban vs. rural areas of the US in the modern era of effective HIV therapy and increased emphasis on HIV testing. Thus, there is need to update prior studies with more recent data on HIV testing frequencies in rural compared to urban areas.

In an effort to inform discussion about HIV testing strategies in rural areas, we analyzed national Behavioral Risk Factor Surveillance System (BRFSS) data with two goals: 1) to determine self-reported HIV testing frequencies in urban compared to rural populations of the US in the modern era of effective HIV therapy, and 2) to determine the types of sites where HIV testing is occurring in urban vs. rural areas.

## Methods

### Data sources and population

This study used publically-available BRFSS data to generate nationally-representative, cross-sectional estimates of the frequency of HIV testing in the US population in 2005 and 2009, according to urban vs. rural residential status. BRFSS is an annual, random-digit-dialed telephone survey of the US civilian, non-institutionalized population aged ≥ 18 years [7]. State-level data are combined and weighted to create a nationally representative sample. Survey response rates during these years ranged from 51% to 53% [8]. We also obtained the non-public use BRFSS data for 2005 that included Federal Information Processing Standard (FIPS) county codes and used these to link data for each respondent with county-based Urban Influence Codes (UIC) developed by the United States Department of Agriculture [9]. This allowed more precise classification of urban vs. rural residence. CDC policy did not allow release of non-public 2009 BRFSS data including county codes, thus preventing linkage to UICs for this year.

### Variables

The primary dependent variables were dichotomous measures indicating whether respondents reported HIV testing in the past 12 months or ever in their lifetime. BRFSS limited questions regarding HIV testing to adults age 18-64 and excluded HIV tests performed during blood donation. Persons with missing HIV-testing data due to refusal to answer questions or uncertainly regarding prior testing (~ 2% of respondents) were excluded. This follows the approach applied in prior reports of HIV testing rates in CDC survey data [10].

Among respondents reporting a prior HIV test, BRFSS also asked "Where did you have your last HIV test: at a private doctor or HMO office, at a counseling and testing site, at a hospital, at a clinic, in a jail or prison, at home, or somewhere else?" We combined responses to create a six-level categorical variable for the type of site where the HIV test occurred: 1) outpatient healthcare setting (private doctor, HMO office, or clinic), 2) hospital, 3) counseling and testing site, 4) home, 5) other, and 6) not sure.

The primary independent variable was a measure of respondent urban vs. rural residence that followed the metropolitan area framework developed by the US Office of Management and Budget in 2003, based on 2000 Census data [2]. The metropolitan area framework is a commonly used measure of urban ("metropolitan") vs. rural ("non-metropolitan") environments in the US. In each year, BRFSS classified respondents as living in the center city of a metropolitan area, in a metropolitan area but not in a center city, or in a non-metropolitan area. For 2005 data, we used UICs linked to county codes to further classify non-metropolitan areas. These codes combined population and commuting data to provide more precise definitions of stages along the urban-rural continuum [9]. Urban influence codes were not available for the state of Alaska.

This allowed creation of a five-level variable describing respondent urban vs. rural residence: 1) metropolitan-center city (the most urban), 2) metropolitan-other (including suburban areas), 3) non-metropolitan-but adjacent to a metropolitan area (UICs 3-7), 4) non-metropolitan-micropolitan (not adjacent to a metropolitan area but with a town/urban cluster of 10,000 residents or greater, UIC 8); or 5) remote non-metropolitan (the most rural, UIC 9-12). It was not possible to link Urban Influence Codes to 2009 data. For analysis of 2009 data we classified respondents simply as metropolitan or non-metropolitan.

Covariates were selected from prior reports of factors associated with HIV testing rates in CDC survey data, including BRFSS and the National Health Interview Study (NHIS) [10,11]. These included self-reported age, gender, race-ethnicity (classified as White, non-Hispanic; Black, non-Hispanic; Hispanic; or other), region of the United States by census criteria (Northeast, South, Midwest, and West), and self-reported presence of HIV risk factors. Respondents were asked whether they had any of the following HIV risk factors in the past year: intravenous drug use, treatment for a sexually transmitted infection, exchange of money or drugs for sex, or anal sex without a condom. Responses were used to create a dichotomous variable indicating self-reported presence of HIV risk factors.

### Analysis

We first examined 2005 BRFSS data, the most recent year for which Urban Influence Codes allowed more precise definition of urban vs. rural residence. We determined demographic characteristics (age, gender, race/ethnicity, and region of residence) and frequency of HIV risk factors among US adults age 18-64 according to urban vs. rural residential status. We next determined the frequencies of lifetime and past year HIV testing in 2005 by metropolitan status. We also determined the type of site where the HIV test occurred according to respondent residential status. Sample weights allowed generation of nationally representative estimates. Categorical measures were compared using chi-square statistics and continuous measures using rank-sum tests. All analyses accounted for the complex survey design using SUDAAN software v. 9 (Research Triangle Park, NC).

We next used 2005 data to fit logistic regression models to determine the association between residential status and HIV testing in the past year after adjusting for possible confounders, including age, gender, race/ethnicity, region, and presence of HIV risk factors. We examined all variables for collinearity (correlation coefficient > 0.5). Models were developed through backwards elimination, beginning with testing for interactions between residence and other variables using a cutoff of P < 0.01 for significance. We fitted two multivariate models: 1) including rural vs. urban residence and demographics (age, race/ethnicity, sex, and region), and 2) including rural vs. urban residence, demographics, and presence of a self-reported HIV risk factor.

In order to determine whether differences in HIV testing rates between urban and rural populations were stable over time, we also examined HIV testing rates according to residential status using 2009 BRFSS data, the most recent year available. As it was not possible to further classify residential status in 2009 according to UIC, we categorized respondents as simply metropolitan ("urban") or non-metropolitan ("rural").

## Results

The 2005 sample included 257,895 respondents who were representative of the entire US population, excluding Alaska. As the residential environment became progressively more rural, respondents were older, more likely to be white, more likely to live in the South or Midwest, less likely to live in the Northeast or West, and less likely to self-report an HIV risk factor (Table 1).

**Table 1:** Characteristics of U.S. Adults Age 18-64 in 2005, by Metropolitan Residence

			**METROPOLITAN**		**NON-METROPOLITAN**		
				
	***p***	***Overall*** **N = 257,895**	***Central City*** **N = 84,492**	***Other*** **N = 92,818**	***Adjacent*** **N = 44,096**	***Micropolitan*** **N = 20,584**	***Remote*** **N = 15,905**
		
***Age, Median (IQR)***	*< 0.001*	39 (29-50)	38 (28-49)	40 (30-50)	41 (29-51)	41 (29-51)	42 (30-52)
***Race/ethnicity, % (SE)***	*< 0.001*						
White, non-Hispanic		68.5 (0.2)	56.8 (0.4)	73.1 (0.3)	82.9 (0.4)	81.4 (0.6)	85.5 (0.6)
Black, non-Hispanic		10.1 (0.1)	14.7 (0.2)	7.4 (0.2)	7.2 (0.2)	5.9 (0.3)	5.4 (0.6)
Hispanic		15.5 (0.2)	21.2 (0.4)	14.2 (0.3)	6.0 (0.3)	7.9 (0.5)	5.6 (0.4)
Other		5.2 (0.1)	6.6 (0.2)	4.7 (0.2)	3.3 (0.2)	4.1 (0.4)	3.1 (0.3)
Unknown		0.7 (0.1)	0.7 (0.1)	0.6 (0.1)	0.6 (0.1)	0.7 (0.1)	0.4 (0.1)
***Sex, % Female (SE)***	*.96*	50.0 (0.2)	50.0 (0.3)	50.0 (0.3)	50.3 (0.5)	50.3 (0.7)	49.8 (0.7)
***Region, % (SE)***	*< 0.001*						
Northeast		18.6 (0.1)	15.4 (0.2)	24.0 (0.2)	15.2 (0.3)	8.5 (0.3)	5.1 (0.3)
South		35.8 (0.1)	36.7 (0.3)	32.2 (0.2)	45.5 (0.4)	33.7 (0.6)	42.1 (0.7)
Midwest		22.3 (0.1)	19.1 (0.2)	21.7 (0.2)	28.1 (0.4)	36.0 (0.6)	38.2 (0.7)
West		23.2 (0.1)	28.8 (0.3)	22.1 (0.3)	11.2 (0.3)	21.8 (0.5)	14.6 (0.5)
***HIV Risk Factor, % (SE)***	*< 0.001*	4.0 (0.1)	4.7 (0.2)	3.7 (0.2)	3.3 (0.2)	3.3 (0.3)	2.9 (0.3)

**Figure 1:**
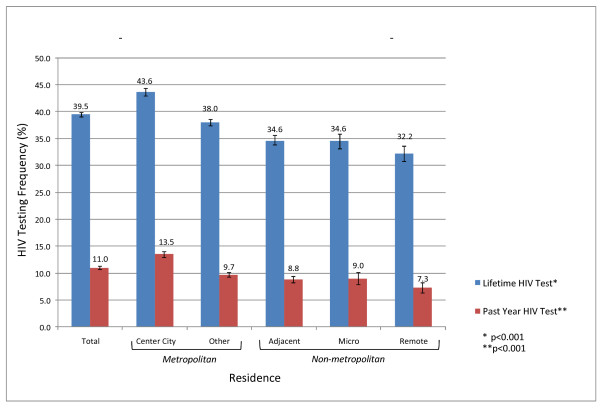
**Lifetime and past-year HIV testing frequencies among US adults age 18-64 in 2005, by metropolitan residence**.

Both lifetime and past-year HIV testing rates varied significantly along the urban-rural continuum (Figure 1). Residents of the United States were progressively less likely to report prior HIV testing as their environment became more rural. Lifetime testing frequencies ranged from 43.6% among persons residing in center cities of metropolitan areas (the most urban areas) to 32.2% among persons in remote non-metropolitan regions (the most rural areas). Past-year testing frequencies ranged from 13.5% to 7.3% in these groups (P < 0.001 for both comparisons).

All variables were significant in the multivariable models and there were no significant interactions between residence and covariates. Model #1 indicated that demographic characteristics substantially confounded the association between rural vs. urban residence and HIV testing, as indicated by attenuation of the effects related to residence (Table #2). As in prior studies, past year HIV testing was more common among younger persons, Blacks, Hispanics, and women [10,11]. Additional adjustment for self-reported HIV risk factors in model #2 led to less substantial change in the association between rural vs. urban residence and HIV testing. After adjusting for demographic characteristics (age, race/ethnicity, sex, region) and self-reported presence of an HIV risk factor, the likelihood of HIV testing in the past year decreased progressively as respondent residence became more rural (adjusted odds ratio for respondents in remote non-metropolitan regions compared to center city of metropolitan areas 0.65, 95% CI 0.57-0.75).

**Table 2:** Univariate and Multivariate Odds Ratios for Past Year HIV Testing among U.S. Adults Age 18-64

	Univariate OR*	Multivariate OR* Model #1 ^†^	Multivariate OR* Model #2 ^‡^
Urban-rural residence			
Metropolitan, center city	Ref	Ref	Ref
Metropolitan, other	0.68 (0.64-0.73)	0.78 (0.73-0.85)	0.81 (0.75-0.87)
Non-metro, adjacent	0.61 (0.56-0.67)	0.75 (0.68-0.83)	0.77 (0.70-0.84)
Non-metro, micropolitan	0.63 (0.55-0.73)	0.78 (0.67-0.91)	0.80 (0.70-0.93)
Non-metro, remote	0.50 (0.44-0.57)	0.62 (0.54-0.73)	0.65 (0.57-0.75)
Age			
18-24	5.46 (4.85-6.15)	5.21 (4.60-5.90)	4.87 (4.30-5.51)
25-34	4.89 (4.40-5.44)	4.61 (4.13-5.16)	4.47 (3.99-5.00)
35-44	2.95 (2.65-3.29)	2.86 (2.56-3.20)	2.82 (2.52-3.16)
45-54	1.60 (1.43-1.80)	1.59 (1.41-1.79)	1.57 (1.39-1.77)
55-64	Ref	Ref	Ref
Race/ethnicity			
White, non-hispanic	Ref	Ref	Ref
Black, non-hispanic	3.90 (3.6-4.2)	3.37 (3.12-3.64)	3.33 (3.08-3.59)
Hispanic	1.94 (1.77-2.13)	1.49 (1.35-1.65)	1.46 (1.32-1.62)
Other	1.43 (1.22-1.70)	1.26 (1.08-1.48)	1.23 (1.05-1.45)
Unknown	1.20 (0.9-1.6)	1.15 (0.85-1.56)	1.15 (0.86-1.55)
Sex, female	1.09 (1.03-1.20)	1.10 (1.05-1.20)	1.10 (1.04-1.20)
Region			
Northeast	Ref	Ref	Ref
South	1.18 (1.10-1.27)	1.01 (0.93-1.09)	1.01 (0.94-1.10)
Midwest	0.71 (0.65-0.77)	0.71 (0.65-0.78)	0.71 (0.65-0.77)
West	0.95 (0.86-1.05)	0.89 (0.80-0.99)	0.88 (0.80-0.98)
HIV risk factor	2.88 (2.54-3.26)	***	2.08 (1.82-2.38)

* OR = odds ratio and 95% confidence interval

† Model #1:residence and demographics

‡ Model #2:residence, demographics, and HIV risk factor

The type of site where the test occurred also varied by respondent residence. In general, rural persons with a prior HIV test were less likely to report testing in an outpatient medical clinic or HIV counseling and testing site, but more likely to report testing in a hospital (Table 3).

**Table 3:** Type of HIV testing site by respondent residence (% of persons reporting test)

			**Metropolitan**		**Non-Metropolitan**		
				
	***p***	***Overall***	***Central City***	***Other***	***Adjacent***	***Micropolitan***	***Remote***
		
**Testing Location**	*< 0.001*						
Outpatient clinic		64.3	66.0	64.0	60.8	60.0	57.8
Hospital		18.4	17.2	18.1	22.6	21.7	24.9
Counseling/testing site		3.3	4.0	3.0	2.1	2.7	2.0
Home		4.0	3.5	4.7	3.9	3.6	4.1
Other		8.7	8.2	8.8	9.5	11.0	10.0
Not sure		1.3	1.1	1.4	1.1	1.0	1.2

**Figure 2:**
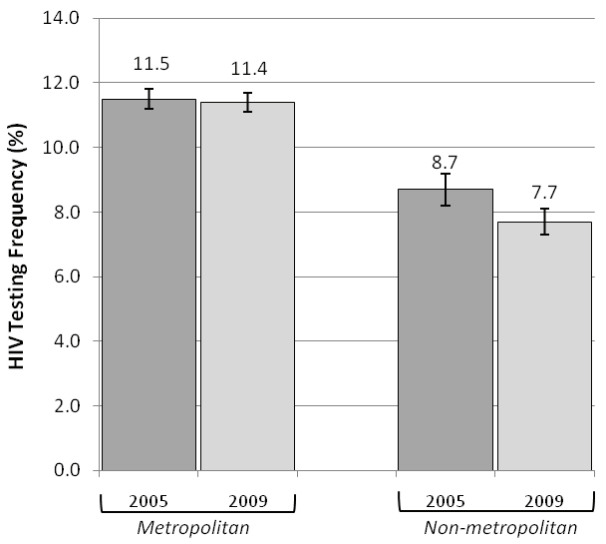
**Frequency of past-year HIV testing in 2005 vs. 2009, by metropolitan residence**.

Past year HIV testing rates did not change substantially between 2005 and 2009 in either urban or rural areas (Figure 2). In the metropolitan (urban) group 11.5% (95% CI 11.2-11.8) reported past-year HIV testing in 2005 vs. 11.4% (95% CI 11.1%-11.7%) in 2009 (P = 0.93). Testing rates actually declined slightly in the non-metropolitan (rural) group; 8.7% (95% CI 8.2%-9.2%) were tested in 2005 vs. 7.7% (95% CI 7.2%-8.2%) in 2009 (P = 0.03).

## Discussion

In this nationally-representative, population-based study of HIV testing frequencies in the United States, we found that the frequency of self-reported HIV testing decreased substantially as the residential environment became progressively more rural. After adjusting for differences in demographics and self-reported HIV risk factors, the odds of HIV testing in the past year were 35% lower among persons living in the most remote rural areas compared to persons in the most urban areas. Rural persons with a prior HIV test were more likely than urban to report testing in a hospital, but less likely in the outpatient setting.

A prior study in the early years of the HIV epidemic in the US also found that rural persons were less likely than urban to report HIV testing [6]. Our results demonstrate that this gap in testing persists in the modern era of effective HIV therapy, when early diagnosis and linkage to care are even more essential. Moreover, recent efforts to increase testing have not impacted the rural-urban gap in testing. Although rural persons with HIV experience barriers to care, prior studies have described effective models for delivering high-quality HIV care in rural settings [12-18]. This accentuates the importance of early testing and diagnosis among rural persons with HIV.

The observation of lower HIV testing rates in rural areas is consistent with published evidence that rural persons are, on average, diagnosed with HIV at a later stage of infection than urban persons [4]. Persons diagnosed at a later stage of HIV infection experience worse outcomes than persons diagnosed early and may unknowingly transmit infection to others [19,20]. Thus, there is pressing need for efforts to promote increased HIV testing and earlier diagnosis in rural areas.

Lower rates of HIV testing in rural areas may result from decreased access to settings where testing occurs, including both healthcare delivery sites and community-based HIV counseling and testing programs. In addition, rural healthcare providers may have less experience in HIV medicine and be less likely to recommend testing [12]. Stigma surrounding HIV infection may make rural persons less likely to seek testing [15]. Such stigma is intensified in rural areas due to a perceived lack of anonymity and fear that privacy will be lost when seeking testing [21,22].

What is the optimal frequency of HIV testing in rural populations in the US? Until recently, the US has pursued a risk-based approach to HIV testing. Prior to 2006, the CDC recommended HIV testing for persons with identified risk factors, including persons with history of intravenous drug use or sexually transmitted infections, men who have sex with men, persons who have exchanged sex for money or drugs, or their sex partners [23]. Under this approach, HIV testing rates would naturally be lower in rural areas, where fewer persons report HIV risk factors. However, using 2005 data when risk-based testing policies were in place, we found that even after adjusting for differences in self-reported HIV risk factors and demographics, rural persons were substantially less likely to report HIV testing than urban persons.

In 2006 the CDC revised its policies and recommended routine, voluntary HIV testing in the healthcare setting for all persons between ages 13 and 64 [24]. This change in policy reflected growing recognition that risk-based HIV testing strategies were inadequate. Despite prior risk-based testing policies, nearly half of persons with newly-diagnosed HIV infection were recognized late in their course and met criteria for Acquired Immune Deficiency Syndrome (AIDS)-an advanced stage of immune compromise-within a year of HIV diagnosis [25]. Many had missed opportunities for HIV testing and earlier diagnosis during prior healthcare encounters, in part due to lack of perceived risk for HIV infection or reluctance to disclose HIV risk factors to providers [5,25]. Routine testing seeks to remove stigma associated with identifying persons with HIV risk factors for testing, which may particularly increase testing among persons who are at risk for infection but who do not report risk factors. The overall goal of routine testing in healthcare settings is to increase the frequency of HIV testing in the overall US population, thus increasing the likelihood that persons in early stages of HIV infection undergo testing and preventing late diagnosis.

The rate of HIV testing did not change substantively in either urban or rural areas between 2005 and 2009, when routine testing policies had been in place for several years. In fact, the frequency of past-year testing declined slightly in rural areas. This indicates that the routine testing recommendation did not meaningfully impact the tendency toward less HIV testing in rural areas. Regardless of whether one views our results from a risk-based or population-based testing perspective, there is a substantial gap in HIV testing rates between urban and rural areas.

The relevance of CDC's routine testing policy to rural areas is unclear. Although modeling indicates that routine testing is cost-effective, with estimates generally less than $70,000 per Quality-adjusted life year (QALY) gained compared to prior testing policies, cost-effectiveness worsens as the prevalence of undiagnosed HIV infection in the local population decreases [26]. Prevalence of undiagnosed HIV infection is generally unknown in rural areas of the US, but is probably often significantly less than 0.1%. This mitigates the public health impact of routine HIV-testing in rural healthcare settings.

On the other hand, cost-effectiveness of routine testing improves as the background rate of HIV testing in the community decreases, leading to HIV diagnosis at a later stage of infection for many persons [26]. It is important to note that cost-effectiveness of routine HIV testing may be favorable in rural areas with low background rates of HIV testing and correspondingly high rates of late HIV diagnosis, even when local prevalence of undiagnosed HIV infection is low. To address this possibility, future studies should use HIV surveillance data, which are now geocoded in many states, and published mathematical models to explore the cost-effectiveness of routine HIV testing across a range of rural healthcare settings.

This study has several limitations. BRFSS relied on self-report of HIV testing and risk factors. Respondents may have had inaccurate awareness or recall of prior HIV testing. Stigma related to HIV infection may have made respondents reluctant to disclose information regarding HIV risk factors in a telephone survey. Disclosure of HIV risk factors is also incomplete during healthcare encounters and revised testing guidelines now emphasize population-based instead of risk-based HIV testing [5]. We therefore believe that the population-based HIV testing frequencies reported here are relevant, even if adjustment for HIV risk-factors was imperfect.

CDC recommendations call for routine HIV testing in the healthcare setting for persons age 13-64, but BRFSS did not include persons under age 18. BRFSS also did not include persons who were homeless, incarcerated, in the military, or in long-term care facilities. These are important populations when considering HIV testing policies.

BRFSS employs random-digit-dialing of land-based telephones. An increasing segment of the US population uses cell phones only, and this population is younger and displays differing health behavior patterns than land-line users [7]. Future studies of HIV testing frequencies would benefit from strategies to sample younger populations at increased risk for HIV infection, such as use of cell-phone and internet-based survey techniques [7]. Finally, this was a cross-sectional analysis. A cohort study analyzing time to HIV testing among rural and urban persons (survival analysis) may provide better estimates of testing discrepancies and avoid biases due to migration and loss to follow up.

## Conclusions

Previous work indicates that rural persons with HIV are more likely than their urban counterparts to be diagnosed at a late stage of infection, suggesting missed opportunities for HIV testing in rural areas. Our results confirm that both overall and risk-factor-adjusted HIV testing rates are lower in rural compared to urban populations in the US. Together, these findings demonstrate a pressing need to develop and implement cost-effective strategies to increase HIV testing, whether risk-based or routine, that are tailored to the unique needs of the rural setting. Potential strategies to increase HIV testing in rural areas include: 1) implementation of nurse-initiated routine testing in general primary care, 2) integration of testing into a range of easily-accessible health services, such as community pharmacies, and 3) use of rapid HIV testing [21,27].

## Competing interests

The authors declare that they have no competing interests.

## Authors' contributions

MO participated in study conception, data analysis, and manuscript preparation. EP participated in study conception and manuscript preparation. All authors read and approved the final manuscript.

## Pre-publication history

The pre-publication history for this paper can be accessed here:

http://www.biomedcentral.com/1471-2458/11/681/prepub

## References

[B1] HIV Surveillance in Urban and Nonurban Areas Centers for Disease Control and Prevention (CDC) http://www.cdc.gov/hiv/topics/surveillance/resources/slides/urban-nonurban/index.htm Last updated Jun-1-2010. Last accessed Feb-1-2011

[B2] Hart LG Larson EH Lishner DM Rural definitions for health policy and research Am J Public Health 2005 95 1149 1155 10.2105/AJPH.2004.042432 15983270 PMC1449333

[B3] Ohl M Tate J Duggal M Skanderson M Scotch M Kaboli P Vaughn-Sarrazin M Justice A Rural residence is associated with delayed care entry and increased mortality among veterans with human immunodeficiency virus infection Med Care 2010 48 1064 1070 10.1097/MLR.0b013e3181ef60c2 20966783 PMC3138500

[B4] Weis KE Liese AD Hussey J Gibson JJ Duffus WA Associations of rural residence with timing of HIV diagnosis and stage of disease at diagnosis, South Carolina 2001-2005 J Rural Health 2010 26 105 112 10.1111/j.1748-0361.2010.00271.x 20446996

[B5] Chou R Huffman LH Fu R Smits AK Korthuis PT Screening for HIV: a review of the evidence for the U.S. Preventive Services Task Force Ann Intern Med 2005 143 55 73 15998755 10.7326/0003-4819-143-1-200507050-00010

[B6] Mainous AG Neill RA Matheny SC Frequency of human immunodeficiency virus testing among rural US residents and why it is done Arch Fam Med 1995 4 41 45 10.1001/archfami.4.1.41 7812475

[B7] Mokdad AH The Behavioral Risk Factors Surveillance System: past, present, and future Annu Rev Public Health 2009 30 43 54 10.1146/annurev.publhealth.031308.100226 19705555

[B8] Office of Surveillance Epidemiology, and Laboratory Services BRFSS-Summary Data Quality Reports Centers for Disease Control http://www.cdc.gov/brfss/technical_infodata/quality.htm Last updated Mar-2-2011. Last accessed Mar-5-2011

[B9] US Department of Agriculture Measuring Rurality: Urban Influence Codes USDA Economic Research Service http://www.ers.usda.gov/briefing/rurality/urbaninf/Last updated 8-8-2007

[B10] Persons tested for HIV--United States, 2006 MMWR Morb Mortal Wkly Rep 2008 57 845 849 18685551

[B11] CDC Number of persons tested for HIV-United States, 2002 MMWR Recomm Rep 2004 1110 1113 15573028

[B12] Cohn SE Berk ML Berry SH Duan N Frankel MR Klein JD McKinney MM Rastegar A Smith S Shapiro MF Bozzette SA The care of HIV-infected adults in rural areas of the United States J Acquir Immune Defic Syndr 2001 28 385 392 11707677 10.1097/00126334-200112010-00013

[B13] Grace C Kutzko D Alston WK Ramundo M Polish L Osler T The Vermont model for rural HIV care delivery: eleven years of outcome data comparing urban and rural clinics J Rural Health 2010 26 113 119 10.1111/j.1748-0361.2010.00272.x 20446997

[B14] Grace CJ Soons KR Kutzko D Alston WK Ramundo M Service delivery for patients with HIV in a rural state: the Vermont model AIDS Patient Care STDS 1999 13 659 666 10.1089/apc.1999.13.659 10743511

[B15] Heckman TG Somlai AM Peters J Walker J Otto-Salaj L Galdabini CA Kelly JA Barriers to care among persons living with HIV/AIDS in urban and rural areas AIDS Care 1998 10 365 375 10.1080/713612410 9828979

[B16] Lahey T Lin M Marsh B Curtin J Wood K Eccles B von Reyn CF Increased mortality in rural patients with HIV in New England AIDS Res Hum Retroviruses 2007 23 693 698 10.1089/aid.2006.0206 17530995 PMC2872149

[B17] McKinney MM Variations in rural AIDS epidemiology and service delivery models in the United States J Rural Health 2002 18 455 466 10.1111/j.1748-0361.2002.tb00910.x 12186320

[B18] Sheth SH Jensen PT Lahey T Living in rural New England amplifies the risk of depression in patients with HIV BMC Infect Dis 2009 9 25 10.1186/1471-2334-9-25 19265529 PMC2670845

[B19] Marks G Crepaz N Senterfitt JW Janssen RS Meta-analysis of high-risk sexual behavior in persons aware and unaware they are infected with HIV in the United States: implications for HIV prevention programs J Acquir Immune Defic Syndr 2005 39 446 453 10.1097/01.qai.0000151079.33935.79 16010168

[B20] Palella FJ Jr oria-Knoll M Chmiel JS Moorman AC Wood KC Greenberg AE Holmberg SD Survival benefit of initiating antiretroviral therapy in HIV-infected persons in different CD4+ cell strata Ann Intern Med 2003 138 620 626 12693883 10.7326/0003-4819-138-8-200304150-00007

[B21] Sutton M Anthony MN Vila C Lellan-Lemal E Weidle PJ HIV testing and HIV/AIDS treatment services in rural counties in 10 southern states: service provider perspectives J Rural Health 2010 26 240 247 10.1111/j.1748-0361.2010.00284.x 20633092

[B22] Zukoski AP Thorburn S Experiences of stigma and discrimination among adults living with HIV in a low HIV-prevalence context: a qualitative analysis AIDS Patient Care STDS 2009 23 267 276 10.1089/apc.2008.0168 19260770

[B23] CDC Revised guidelines for HIV counseling, testing, and referral MMWR Recomm Rep 2001 50 1 62 11718472

[B24] Branson BM Handsfield HH Lampe MA Janssen RS Taylor AW Lyss SB Clark JE Revised recommendations for HIV testing of adults, adolescents, and pregnant women in health-care settings MMWR Recomm Rep 2006 55 1 17 16988643

[B25] Missed opportunities for earlier diagnosis of HIV infection--South Carolina, 1997-2005 MMWR Morb Mortal Wkly Rep 2006 55 1269 1272 17136020

[B26] Paltiel AD Weinstein MC Kimmel AD Seage GR Losina E Zhang H Freedberg KA Walensky RP Expanded screening for HIV in the United States--an analysis of cost-effectiveness N Engl J Med 2005 352 586 595 10.1056/NEJMsa042088 15703423

[B27] Anaya HD Hoang T Golden JF Goetz MB Gifford A Bowman C Osborn T Owens DK Sanders GD Asch SM Improving HIV screening and receipt of results by nurse-initiated streamlined counseling and rapid testing J Gen Intern Med 2008 23 800 807 10.1007/s11606-008-0617-x 18421508 PMC2517869

